# Autologous Platelet-Rich Plasma (PRP) in Infertility—Infusion versus Injectable PRP

**DOI:** 10.3390/jpm13121676

**Published:** 2023-11-30

**Authors:** Ioana Alexandra Zaha, Anca Huniadi, Florin Bodog, Luana Seles, Mihaela Cristina Toma, Laura Maghiar, Erika Szulay-Bimbo, Alin Bodog, Liliana Sachelarie, Mihai Florea, Liana Stefan

**Affiliations:** 1 Faculty of Medicine and Pharmacy, University of Oradea, 1st December Square 10, 410073 Oradea, Romania; drzahaioana@gmail.com (I.A.Z.); ancahuniadi@gmail.com (A.H.); fbodog@gmail.com (F.B.); luana.seles@yahoo.com (L.S.); criss650@gmail.com (M.C.T.); lauratodan@yahoo.ro (L.M.); bszera@gmail.com (E.S.-B.); lianaantal@gmail.com (L.S.); 2 A Calla—Infertility Diagnostic and Treatment Center, Constantin A. Rosetti Street, 410103 Oradea, Romania; mihai_drf@yahoo.com; 3 Pelican Clinical Hospital, Corneliu Coposu Street 2, 410450 Oradea, Romania; 4 Oradea County Hospital, Gheorghe Doja Street 65-67, 410169 Oradea, Romania; 5 Department of Prelinical Discipline, Apollonia University, 700511 Iasi, Romania

**Keywords:** thin endometrium, PRP, frozen embryo transfer, endometrial thickness, IVF

## Abstract

(1) Background: During IVF (in vitro fertilization) procedures, endometrial thickness has a significant role in the success of pregnancy outcomes for embryo transfers. Endometrial thickness, a crucial component of endometrial receptivity, is a contentious issue. The regenerative properties of PRP have been shown in recent research to have positive effects on the endometrium. PRP increases the pregnancy rate in IVF patients with thin endometrium and recurrent implantation failure. In order to demonstrate the efficacy of PRP therapies, this work compares the administration of injectable and infusible PRP during endometrial preparation. (2) Methods: This prospective single-arm control study was conducted at an IVF center in Oradea, Romania. This study included 50 patients; 27 were included in the group with Injectable PRP and 23 in the group with Infusible PRP. The outcome was compared between the two groups, with the primary outcome being the endometrial thickness after the PRP infusion or injection and the secondary outcome being the pregnancy rate in both groups. (3) Results: Patients who were treated with Injectable PRP had a higher pregnancy rate. An improvement in the quality of the endometrium, in terms of thickness, was also observed in the patients who were injected with PRP. (4) Conclusions: Compared to PRP infusions inside the uterus, sub-endometrial PRP injections in frozen embryo transfer methods have a greater pregnancy rate.

## 1. Introduction

Platelet-rich plasma, or PRP, is used in a variety of injectable and infusible methods for treating thin endometrium, recurrent implantation failure (RIF), and refractory endometrium in infertility cases.

Following an embryo transfer, synchronization of the endometrium and the embryo is crucial for achieving a pregnancy. The blastocyst is largely responsible for a significant portion of repeated implantation failure; however, other diseases, such as thin endometrium, should also be taken into consideration. Research has indicated that fewer pregnancies occur when the endometrium is less than 7 mm prior to progesterone intake in embryo transfer protocols, as opposed to when it is thicker than 7 mm [1].

Even though a lot of authors maintain a minimum endometrial thickness of 7 mm, there is ongoing disagreement over this because current research indicates that endometrial thickness does not correlate with live births (LBR) of zero [2]. However, as the moment of trigger should occur during the best window for endometrium receptivity rather than the homogeneous, hyperechogenic endometrium, endometrial pattern is a significant predictor [3]. According to one study, people over 35 years of age should have an endometrial thickness of more than 7 mm since this is associated with a higher pregnancy rate due to the “aging of the endometrium”, while thickness does not correspond with the pregnancy rate in patients under 35 [4]. Though in frozen embryo transfer cycles, prolonged estradiol exposure or adjuvant treatments such as low-dose aspirin, vaginal administration of sildenafil citrate, pentoxifylline, vitamin E, and tamoxifen are commonly used, they have not proven a certain effect on endometrial thickness or pregnancy rate [5].

For the subject at hand, the thin endometrium, the first use of PRP was in 2015 by Chang et al. and is being used in many medical fields such as orthopedics and plastic surgery, but it is a novel add-on in selected cases during IVF with promising results [6]. PRP contains insulin-like growth factors 1 and 2, epidermal growth factors that promote healing, and by centrifugation and separation with the removal of red blood cells, the concentration of growth factors is 5 to 10-fold higher [7]. Recent studies underline the beneficial effects of PRP on the endometrium due to the cytokine’s regeneration properties [8]. Being rich in cytokines and growth factors and having regenerative effects but also improving endometrial receptivity, PRP can be used easily, and it is an innovative procedure along with the standard endometrial preparation [9].

PRP is used in in vitro fertilization for patients with recurrent implantation failure, and a thin endometrium improves the pregnancy rate [10]. In women with thin endometrium, the literature has shown that endometrial thickness increases and chemical and clinical pregnancy rates increase after autologous PRP therapy. In women with low ovarian reserve, intraovarian autologous PRP therapy increased anti-Müllerian hormone (AMH), decreased follicle-stimulating hormone (FSH), and tended to increase clinical and live birth rates. This trend was also seen in women with recurrent implantation failure after PRP treatment [11].

As stated, the two main components that are intensely studied are the endometrium and the embryo, and having one variable virtually excluded is a big step. On this note, the euploid embryo transfer changes the percentage and improves live birth rates. In embryo-transfers conducted with euploid embryos, Ata et al. concluded that even with a 3–4 mm thickness, the LBR was like the values of 7–8 mm and above [12].

PRP uses the autologous plasma of the patient and has no side effects and no risk of contacting diseases or having an allergic reaction. The uterine infusion is already in practice with good results, and the injection type of technique is showing more interest. For the intrauterine infusion, the amount of plasma is administered in the uterine cavity through a fine catheter several times in the cycle of frozen embryo preparation, with a higher pregnancy rate in women with thin endometrium [13].

The article by Efendieva et al. outlines a procedure where PRP is injected into the sub-endometrial layer using hysteroscopy, resulting in an augmentation of endometrial thickness and an elevated pregnancy rate during the embryo transfer cycle [14]. However, it is worth noting that there is a scarcity of research that directly compares different PRP treatment techniques (infusion versus injection) specifically during embryo transfer in the context of IVF. Furthermore, only a limited number of studies in the existing literature have explored the injection of PRP into the sub-endometrial layer using methods other than hysteroscopy, such as ultrasound-guided injection.

The aim of this paper is to highlight the effectiveness of PRP treatments through a comparative study of injectable and infusible treatments during IVF procedures for certain pathologies, especially the thin endometrium.

## 2. Materials and Methods

This prospective single-arm controlled study was carried out at an IVF center in Oradea, Romania, spanning a duration of two years. This study received approval from the Ethics Committee under the reference number 528/2021 and was conducted at the Calla—Infertility Diagnostic and Treatment Center.

**Figure 1:**
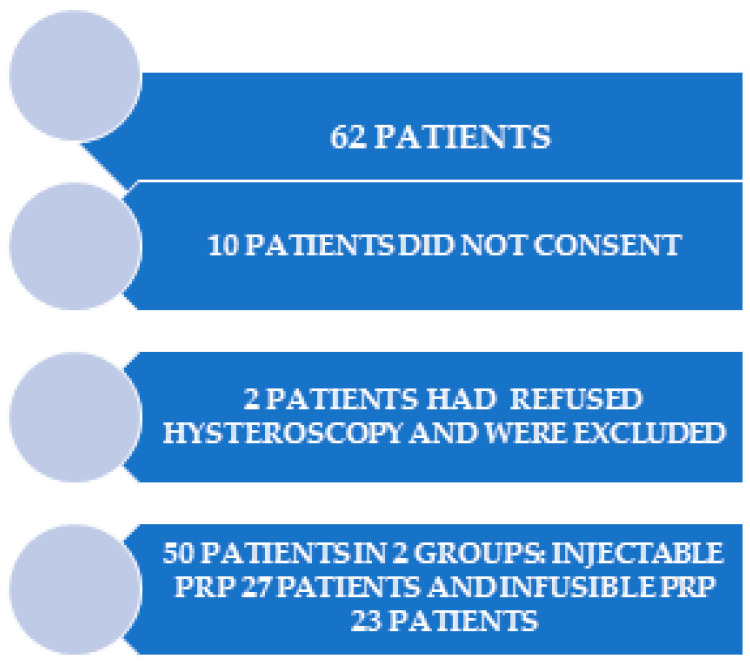
Work flow.

A total of 50 patients were enrolled in this study, with 27 patients included in the group receiving Injectable PRP and 23 patients included in the group receiving Infusible PRP, as illustrated in Figure 1.

Inclusion criteria: patients undergoing an IVF procedure that have at least one top-quality embryo, that accept and sign to be enrolled in this study, having an hysteroscopy before embryo transfer, and a negative test for chronic endometritis. In the case of intrauterine infusion, it should be noted that patients were included if they had experienced at least one cancellation during the preparation for frozen embryo transfer. This cancellation occurred due to their inability to achieve an endometrial thickness of 7 mm even when administered the maximum estradiol dose of 9 × 2 mg daily (ESTROFEM^®^, Novo Nordisk, Bagsværd, Denmark). Additionally, these patients received adjuvant therapy consisting of sildenafil (50 mg daily) (SILDENAFIL^®^, Actavis, Dublin, Ireland), pentoxifylline (400 mg daily) (Pentoxifilina SR^®^, Zentiva, Prague, Czech Republic), and low-dose aspirin (150 mg daily) (ASPENTER^®^, Terapia, Cluj, Romania). Exclusion criteria: acute inflammatory disease, pelvic cancer, intrauterine adhesions (Asherman syndrome), patients refusing the treatment or the hysteroscopy, submucosal fibroma, or endometrial polyps.

Endometrial thickness was assessed using a transvaginal ultrasound probe with a frequency range of 5 to 9 MHz (Voluson E10, GE Healthcare, Chicago, IL, USA). Measurements were taken along the longitudinal axis of the uterus at its thickest point using a vaginal probe.

The patients were divided into two groups: the infusible group and the injectable PRP group.

The infusible PRP group included 23 patients that had the embryo-transfer carried out on the estradiol preparation protocol with an intrauterine PRP infusion of 3 doses during the same embryo-transfer preparation protocol.

The injectable PRP group included 27 patients that had the embryo transfer carried out under the same estradiol preparation protocol with ultrasound-guided PRP sub-endometrial injection in the same cycle.

Primary outcome: increasing the EMT as close as 7 mm on the day of progesterone supplementation.

Secondary outcomes: clinical pregnancy rate; comparing the effects of the two types of treatment on the endometrial thickness.

### 2.1. Endometrial Preparation

In the PRP infusible group, the patient’s assessment began on the second day of their menstrual cycle, involving a transvaginal ultrasound evaluation. Starting on the second day, the patients commenced a regimen of 2 mg of estradiol, taken three times daily. After 7 days, another ultrasound assessment was conducted to measure the endometrial thickness (EMT), and the estradiol dosage was increased to 6 tablets daily. This regimen continued until days 15–18 of the preparation, with EMT measurements taken every 3 days. The first uterine PRP infusion was performed under ultrasound guidance on the 7th day of endometrial preparation. After 2–3 days, the endometrial thickness was reevaluated, and the estradiol dosage was further increased to 9 tablets daily. The second PRP intrauterine infusion took place on the 12th day of estradiol administration, and the third PRP infusion was administered on the day of progesterone supplementation.

For the PRP injection group, the preparation also began on day 2, similar to the infusible group. On the 7th day of endometrial preparation, a single sub-endometrial injection of PRP was performed, guided by ultrasound, and administered under analgo-sedation. This procedure was carried out using a transvaginal probe attachment with a Wallace^®^ Dual Lumen Oocyte Recovery System, delivering 17 g of 2 mL of PRP into the anterior wall and 2 mL into the posterior wall.

Before the day of progesterone supplementation, endometrial thickness was assessed in both groups, and progesterone levels were checked for all patients, requiring levels to be below 1 ng/mL. Progesterone was administered at a dosage of 1200 mg daily via intravaginal administration (UTROGESTAN^®^ 200 mg, Besins Healthcare, (London, UK) Ltd. Medicines) and 25 mg daily via intramuscular injection (PROLUTEX^®^ 25 mg, Goodlife Pharma, (Hatton, UK) Ltd. Medicines). On the day of embryo transfer, progesterone levels were checked again, with a minimum value of >10.6 ng/mL required.

Embryo transfer was performed under transabdominal ultrasound guidance using Cook Medical’s Guardia™ Access Nano. After 10 days, a beta HCG (human chorionic gonadotropin) test was conducted using maternal serum, and medication was continued. A transvaginal ultrasound was scheduled for 4 weeks after embryo transfer to confirm clinical pregnancy.

### 2.2. PRP Preparation

**Figure 2:**
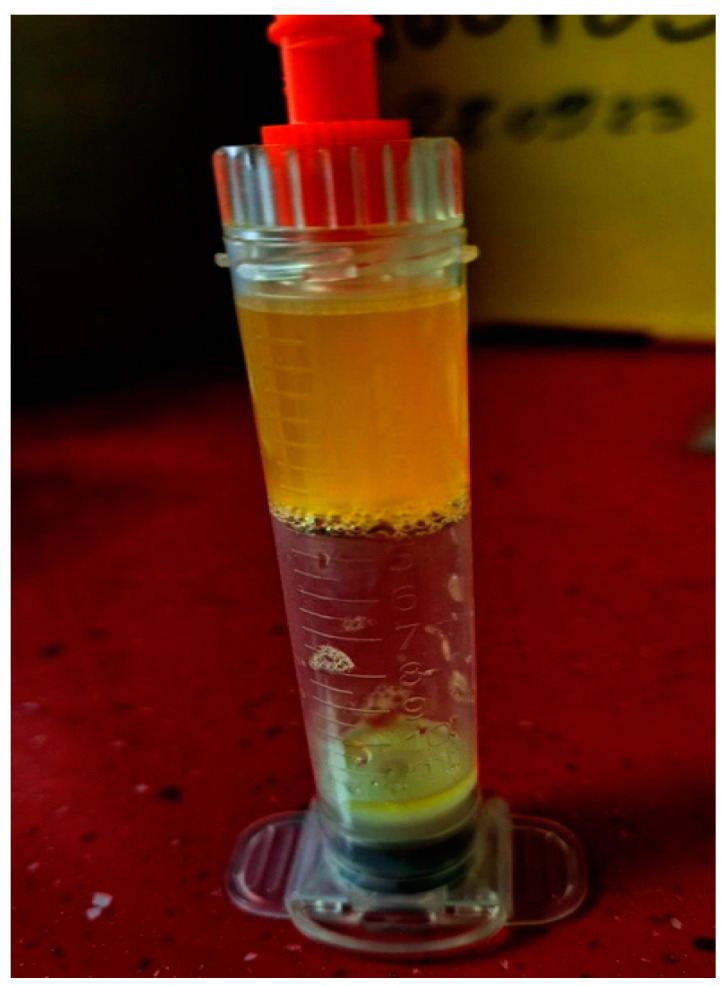
Separation of growth factors and platelets.

The blood is collected by venipuncture in a special tube of 8.6 mL containing 3.8% citrate solution as the anticoagulant from the kit (Endoret^®^ KIT 19). After the venipuncture, the tube needs to be gently inverted four to six times. In a maximum of one hour after the venipuncture, the tube is centrifuged with Endoret^®^ (PRGF^®^) centrifuge System V in 8 min of one cycle. The blood separates into three layers. The yellow layer is the plasma rich in growth factors, which contains platelets; the buffy coat has leukocytes just above the red blood cells; and the third layer is the red cell layer on the bottom of the tube (Figure 2).

The total volume of plasma obtained after centrifugation is contingent on the patient’s hematocrit levels. To extract the plasma, the plasma transfer device should be positioned just beneath the surface of the plasma, typically about 1–2 mm beneath the surface. With care, the plasma fraction is drawn into a 3 mL syringe, excluding any leukocytes or erythrocytes. Subsequently, the plasma is activated by adding a precise amount of endoret activator, specifically 0.02 mL per milliliter of plasma. The contents of the syringe are gently agitated to ensure thorough mixing. The activated plasma is then maintained at a temperature of 37 °C for a minimum of 40 min. Once the plasma has coagulated and undergone retraction, the supernatant is filtered by applying pressure with the plunger, effectively separating it from any remaining components.

### 2.3. Statistical Analysis

Statistical analysis was conducted utilizing SPSS version 26.0 (IBM Corp., Armonk, NY, USA). Continuous variables are reported as means ± standard deviations for normally distributed data, and differences before and after infusion and injectable PRP were compared using the Student’s *t*-test.

## 3. Results

It has been noted that there are no statistically significant differences between the groups regarding the basic characteristics: age, environment, BMI (body mass index), cause of infertility, and duration of infertility (*p* > 0.01) (Table 1).

### 3.1. Before PRP Treatment

The groups had the values of the IVF parameters as shown in Table 2. The endometrial thickness had a mean value of 6.8 mm in our study before PRP administration. Most patients had a mean value of one single embryo-transfer due to the high cancellation rate because of the thin endometrium pathology. Testosterone levels were in the normal range for almost all the patients.

There are no statistically significant differences between the groups (*p* > 0.01), as shown in Table 3. The endometrial mean value was almost the same for both groups, infusible and injectable PRP, before the treatment.

### 3.2. After Treatment

The values of the parameters of the group after the infusion PRP and injectable PRP are given in Table 4.

A significant statistical difference is observed between the two groups (*p* < 0.01). Patients who were treated with injectable PRP had a higher pregnancy rate. An improvement in the quality of the endometrium, in terms of thickness, was also observed in the patients who were injected with PRP (*p* < 0.01) (Table 5). Even in the case of SET, an improvement is observed in the case of the injectable PRP group.

**Figure 3:**
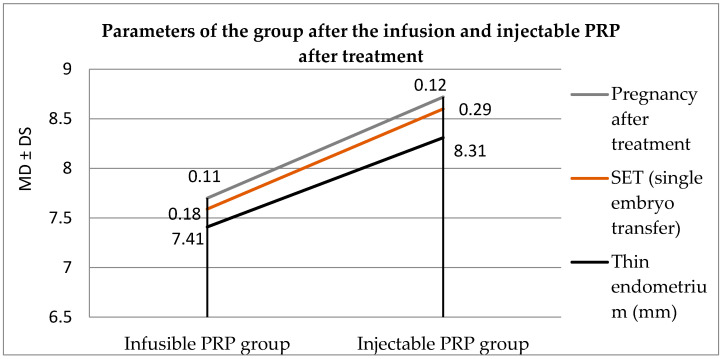
Parameters of the groups after treatment.

It can be seen from Figure 3 that the values of the studied parameters have higher, better values in the case of the group with injectable PRP.

The use of injectable PRP has demonstrated a favorable impact on promoting endometrial proliferation and enhancing both embryo implantation and clinical pregnancy rates in women with thin endometrium. Injecting PRP into the subendometrial layer, despite the need for anesthesia, delivers the substance directly to its targeted site of action. This layer plays a pivotal role in determining the regeneration of the endometrium. The notable improvements in endometrial thickness following PRP treatment strongly suggest its capacity to enhance endometrial receptivity, ultimately resulting in improved outcomes in IVF procedures.

There is a really statistically significant difference between the control group and the group where injectable PRP was used (*p* < 0.01). The values show that the treatment with injectable PRP is much more effective, with a high success rate in obtaining results compared to infusible PRP. We can say that of the treatments applied, the most efficient was the one in which injectable PRP was used.

The pregnancy rate is 38% in the injection group versus 21% in the infusion group.

## 4. Discussion

The objective of this study was to establish the efficacy of injectable PRP compared to infusible PRP in patients with thin endometrium.

In our study, there were no statistically significant differences observed between the groups concerning age, environmental factors, BMI, and the duration of infertility. However, it is noteworthy to mention some relevant findings from previous studies: A study by Tian H et al. revealed a direct relationship between endometrial thickness and ongoing pregnancy rates in women aged over 35 years. Specifically, for each additional millimeter of endometrial thickness, the ongoing pregnancy rate increased by 12% [15].

When it comes to patients with a modified BMI, particularly those who are overweight or obese, recent research suggests that endometrial thickness can be influenced by various factors. Importantly, the BMI alone does not appear to impact endometrial thickness when transferring euploid embryos [16].

In our study, the mean duration of infertility was 5.2 years in the injectable group and 3.4 years in the infusible group. Recent data indicates that in cases of non-male factor infertility, the fertilization rate and pregnancy rate do not seem to be significantly affected, even if the duration of infertility exceeds 5 years. However, for women with specific causes of infertility, such as those related to female factors, a duration of infertility exceeding 4.8 years may lead to a lower fertilization rate in procedures like ICSI (Intracytoplasmic Sperm Injection) [17]. The efficacy of PRP in the treatment of thin endometrium during IVF is rising with more and more studies that prove the benefits of the therapy. Conventional hormone preparation protocols do not always achieve the desired endometrial thickness (over 7 or 8 mm minimum), and adjuvant therapies are needed. Some cases with thin endometrium have a subsequent cause, but there is a percentage of cases that have an unknown etiology, and studies have shown that under the value of 8 mm, an increase in obstetrical adverse outcomes such as preterm delivery can occur [18]. Adjuvant therapy such as tamoxifen proved an increase in endometrial thickness but not in the pregnancy rate [18]. Another type of adjuvant therapy would be sildenafil-citrate use, which is associated with a higher pregnancy rate and not so much with endometrial thickness but with the receptivity and improved vascularization of the endometrium [19,20]. The classic hormonal therapy endometrial preparation in the frozen embryo transfer cycles (FET cycles) has the same pregnancy rate under 7 or 14 days of exposure to estradiol with similar estradiol levels, according to Racca A. et al. [21]. Having these results in mind, adjuvant therapy is needed.

The infusion of PRP in the uterine cavity with the activation of thrombocytes by adding calcium chloride before instillation has a higher pregnancy rate in FET cycles and is an add-on that should be taken into consideration for patients with endometrial pathology [22]. Also, infusible PRP has a positive effect on improving the embryo implantation rate and clinical pregnancy rate for women with thin endometrium compared to conventional hormonal therapy [13]. Because of the many methods of preparation and administration of PRP during embryo-transfers, a clear protocol is needed so the results can be compared as best as possible without taking many variables into consideration.

In a recent study, Zargar et al. found that in the process of randomizing patients, the study group that had the infusible PRP for achieving better IVF results had a smaller but not statistically significant overall IVF success rate than the control group, which consisted of patients that did not have the infusible endometrial PRP [23,24,25,26,27,28].

A study by Tandulwadkar et al. found an increase in endometrial thickness of even 2.2 mm after the infusion of PRP [24]. Furthermore, Eftekhar et al. had an increase in endometrial thickness from 6.09 to 8.67 mm under the PRP infusion [25]. Also, Kusumi et al. had an increase in the endometrium of 0.7 mm and a pregnancy rate of 15.6%, leading to the conclusion that the PRP not only influences the thickness but also the quality of the endometrium [28]. Dogra et al. report in an interventional prospective study that PRP infusion in 20 women increased endometrial thickness in both fresh and frozen cycles with no difference in pregnancy rate in fresh or frozen cycles [26].

In a study conducted by Agarwal et al., they investigated the sub-endometrial injection of PRP guided by hysteroscopy for patients with thin endometrium during IVF treatment. Their findings indicated an improvement in endometrial thickness. Among the 32 patients with refractory thin endometrium, 10 of them achieved clinical pregnancies, and 2 had biochemical pregnancies as a result of this intervention [24].

Compared to the hysteroscopic technique, the sub-endometrial injection of PRP proposed in this study offers several advantages. It is a less invasive procedure that can be performed within the same cycle as the IVF treatment. The intervention has a shorter duration and causes less discomfort for patients.

PRP has the capacity to express important factors like Leukemia Inhibitory Factor (LIF) and Vascular Endothelial Growth Factor (VEGF), which play crucial roles in endometrial receptivity. These markers have been identified in the biopsies of women who experienced infertility and had issues related to their endometrium and/or uterine function [27,28,29]. PRP contains leukocytes, which contribute to endometrial healing. Through sub-endometrial injection, PRP may aid in cases of thin endometrium or damaged endometrium by promoting increased vascularity [30].

Comparing the infusion with the injection of PRP, the endometrial thickness was higher (0.9 mm) in the injection group. In a study by Saravanan et al., the mean endometrial thickness after injecting the PRP in the sub-endometrial layer increased by 0.9 mm compared to the control group, but 37 patients also had a decrease in endometrial thickness after the procedure [26]. In women with thin endometrium, the PRP injection in the sub-endometrial area, according to a study conducted by Cakiroglu Y. et al., had an implantation rate of 17% [31].

In our study, infusible and injectable PRP administration had a higher pregnancy rate, according to the literature. For example, Abduljabbar et al. found a clinical pregnancy rate of 34.3% in the PRP administration compared to 14.3% in the control group [28]. Infusible is easier conducted and requires no anesthesia, and injectable PRP is a slightly invasive procedure with general intravenous anesthesia, which could be discomforting for the patients. We chose the ultrasound-guided injection and not the hysteroscopic one because we wanted to perform the procedure in the same cycle as in the case of the infusible one, which is not possible with hysteroscopic administration.

PRP may act from an immunological point of view and could mediate the synchronization between the embryo and the endometrium in the implantation window by decreasing inflammatory cytokines [32]. Thin endometrium is associated with pregnancy complications such as miscarriage or recurrent implantation failure [33].

Besides endometrial quality and thickness, blastocyst quality is a key factor. For euploid embryos transferred according to age, body mass index, natural cycles, and hormonal preparation cycles, the endometrial thickness was not an independent factor in the live birth rate, and not achieving the 7 mm endometrial thickness and canceled cycles is not justifiable [29,30,31,32,33,34].

Further, PRP is being used in premature ovarian failure and diminished ovarian reserve with promising results. Diminished ovarian reserve, as well as hyperandrogenism and PCOS, are directly linked to thin endometrium or refractory thin endometrium.

In an observational study, Fraidakis et al. had 469 women with premature ovarian failure injected with 2–4 mL of PRP in each ovary with a concentration of platelets of 900.000/µL, and ovarian markers improved in months 3 and 4 after the procedure. PRP increases AMH in poor responders, and BMI had no effect on the results of PRP injections or IVF outcomes for these patients [7].

Studies in the existing literature focused on thin endometrium have consistently indicated the beneficial effects of platelet-rich plasma (PRP) therapy, even though a significant increase in endometrial size is not always achieved. While the exact mechanisms through which PRP enhances implantation are not fully understood, it is postulated that PRP has a positive impact on various cell types within the endometrium, including stromal mesenchymal stem cells, endometrial fibroblasts, endometrial epithelial cells, and marrow-derived stem cells.

The standardization of endometrial preparation is crucial, and our research has achieved this by employing specialized kits to ensure consistency. This approach resulted in the collection of an average of 4–5 mL of plasma, with at least 2 mL injected into both the anterior and posterior uterine walls. It is worth noting that there is currently no consensus in the medical community regarding the optimal volume of PRP, the number of activated platelets per unit volume, the volume of blood to be collected, or the platelet count in the initial sample [7,10,20].

Importantly, PRP is considered a safe technique with no associated side effects, as it involves using the patient’s own blood sample. There are no inherent risks, and the procedure is non-mutagenic in nature.

A significant advantage of our study is the comparison between PRP infusion and injectable PRP procedures within groups that are administered the same medication for embryo transfer. Both methods represent effective ways of administering PRP to women undergoing IVF with thin endometrium or other uterine-related pathologies.

However, it is important to acknowledge this study’s limitations, including the relatively small number of patients involved, as it is a single-center study [12,34,35].

## 5. Conclusions

The injectable, targeted, ultrasound-guided administration of activated PRP in the basal layer of the endometrium represents a promising therapeutic novelty in infertility complicated by thin endometrium, with superior results in pregnancy rate to the non-invasive administration by intrauterine instillation.

## Figures and Tables

**Table 1:** Baseline characteristics of the groups: infusion PRP group and injection PRP group.

Baseline Characteristics of the Group	Infusible PRP Group	Injectable PRP Group	*p* Value
Age of patients (years) MD ± DS	39.16 ± 3.12	37.26 ± 2.32	0.0623
Environment (%)			
Urban	17 (73.91%)	21 (77.77%)	0.0521
Rural	6 (26.08%)	6 (22.23%)	0.0734
BMI (kg/m^2^) MD ± DS	26.2 ± 3.6	24.5 ± 5.8	0.0321
Infertility duration (years) MD ± DS	5.2 ± 1.8	3.4 ± 1.4	0.0542

**Table 2:** Parameters of the patients before PRP treatment.

Parametres	MD ± DS
Thin endometrium (mm)	6.8 ± 1.82
SET (single embryo transfer)	1 ± 1.18
Testosterone (ng/mL)	0.795 ± 0.82
Pregnancy before PRP treatment	0 ± 0.48

**Table 3:** Parameters of the groups: infusion PRP group and injection PRP group.

Parametres	Infusion PRP MD ± DS	Injectable PRP MD ± DS	*p* Value
Thin endometrium (mm)	6.4 ± 1.42	6.2 ± 0.42	0.0321
SET (single embryo transfer)	1 ± 1.12	1 ± 0.12	0.0426
Testosterone (ng/mL)	0.745 ± 0.32	0.7653 ± 0.34	0.0332
Pregnancy	0 ± 0.28	0 ± 0.20	0.0432

**Table 4:** Parameters of the group after the infusion of PRP and injectable PRP.

Parameters	Infusion PRP MD ± DS	Injectable PRP MD ± DS
Thin endometrium (mm)	7.41 ± 0.81	8.31 ± 0.32
SET (single embryo transfer)	2 ± 1.18	2 ± 9.12
Testosterone (ng/mL)	0.785 ± 0.62	0.873 ± 0.44
Pregnancy after PRG treatment	0 ± 0.3	0.4 ± 0.1

**Table 5:** Comparisons of parameters between the infusible PRP group and injectable PRP group.

Parameters	SEM (Standard Error of the Mean)		*p* Value
	Infusible PRP Group	Injectable PRP Group	
Thin endometrium (mm)	0.117	0.114	0.0001
SET (single embryo transfer)	0.18	0.29	0.0002
Pregnancy after treatment	0.110	0.120	0.0001

## Data Availability

The data presented in this study are available on request from the corresponding author.
